# Individual variation explains ageing patterns in a cooperatively breeding bird, the long‐tailed tit *Aegithalos caudatus*

**DOI:** 10.1111/1365-2656.13741

**Published:** 2022-05-24

**Authors:** Mark Roper, Nicole J. Sturrock, Ben J. Hatchwell, Jonathan P. Green

**Affiliations:** ^1^ Department of Zoology University of Oxford Oxford UK; ^2^ Ecology and Evolutionary Biology, School of Biosciences University of Sheffield Sheffield UK

**Keywords:** ageing, cooperative breeding, demography, helping, inclusive fitness, life history, senescence

## Abstract

1. Alloparental care in cooperatively breeding species may alter breeder age‐specific survival and reproduction and subsequently senescence. The helping behaviour itself might also undergo age‐related change, and decisions to help in facultative cooperative breeders are likely to be affected by individual condition.
2. Helpers in long‐tailed tits *Aegithalos caudatus* assist relatives after failing to raise their own brood, with offspring from helped nests being more likely to recruit into the breeding population.
3. Using data collected over 25 years, we examined the age trajectories of survival and reproduction in adult long‐tailed tits to determine how these were affected by the presence or absence of helpers and how helper behaviour changed with age.
4. There was evidence for increased reproductive performance with breeder age, but no effect of age on the probability of survival. We found no evidence of significant senescent decline in survival or reproductive performance, although individuals accrued less inclusive fitness in their last year of life. Lifetime reproductive success was positively related to both reproductive life span and body mass. Within a season, breeders that were assisted by helpers enjoyed greater reproductive success through enhanced offspring recruitment in the following year. We found no evidence that age affected an individual's propensity to help, or the amount of indirect fitness accrued through helping.
5. We found a positive correlation between life span and multiple components of reproductive success, suggesting that individual variation in quality underpins age‐related variation in fitness in this species. Helping decisions are driven by condition, and lifetime inclusive fitness of immigrants was predicted by body mass. These findings further support individual heterogeneity in quality being a major driver for fitness gains across the life course of long‐tailed tits.

## Graphical abstract

How does the presence or absence of helpers affect the age trajectories of survival and reproduction in cooperatively breeding adult long‐tailed tits? Using data collected over 25 years, the authors perform the first explicit test of senescence in helper behaviour.

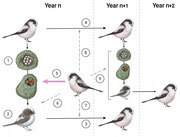

## 1 INTRODUCTION

Senescence is the decline in physiological condition with age that leads to an increased risk of mortality and/or reduced reproductive success after reaching maturity (Medawar, 1952). Although once thought unlikely to be observed in nature, there is now widespread evidence for senescence in wild populations (Gaillard & Lemaître, 2020; Nussey et al., 2013). Studies have also unearthed a diversity of ageing patterns (Jones et al., 2014) that includes negligible (Finch, 1990) or even negative (Vaupel et al., 2004) senescent life histories, where risks of mortality remain constant or decline with age, and reproductive success remains constant or increases with age. Senescence has classically been explained as a result of the declining strength of natural selection with age (Hamilton, 1966; Medawar, 1952; Williams, 1957) and the subsequent trade‐offs between early and late‐life performance that will often favour the former (Kirkwood, 1977). Given the diversity of ageing patterns observed in wild populations, however, the key question to address now is why the decline of the strength of selection with age seems more prominent in some species, and less so or even absent in others. Critically, there is growing theoretical (e.g. Lee, 2003) and empirical (e.g. Hammers et al., 2019) evidence that sociality may contribute significantly to explaining this variation in ageing patterns.

The general relationship between sociality, longevity and ageing appears complex and not unidirectional (Lucas & Keller, 2020). On the one hand, a species' propensity to be social is likely to be driven somewhat by age‐related patterns of survival and reproduction. Indeed, long life with high adult survival and overlapping generations, for example, can provide greater opportunities for individuals to interact, and thus facilitate the evolution of cooperation (Arnold & Owens, 1998; Downing et al., 2015; Ross et al., 2015; Taylor & Irwin, 2000). On the other hand, sociality itself may alter selection gradients on age‐specific survival and reproduction, and therefore the pattern of senescence (Bourke, 2007; Lee, 2003). Most current empirical evidence that sociality can impact senescence comes from studies of cooperative breeders. In cooperatively breeding species, helpers are individuals that aid the rearing of offspring that are not their own. This alloparental care may allow breeders to reduce their current reproductive investment, reproduce at higher rates for longer proportions of their life span and enjoy increased survival (‘load lightening’ – Crick, 1992). This load lightening in turn may delay or even mask senescence (Lucas & Keller, 2020).

Recent evidence supports the idea that load lightening can delay senescence in cooperative breeders (Downing et al., 2021). Studies in facultatively social species such as the Seychelles warbler *Acrocephalus sechellensis* and the Alpine marmot *Marmota marmota* have demonstrated that breeders that receive help experience slower mortality (i.e. actuarial) senescence (Berger et al., 2018; Hammers et al., 2019). Such species‐specific studies have, however, tended to focus on reproductive (Russell et al., 2007; Sharp & Clutton‐Brock, 2010) or actuarial senescence (Berger et al., 2018; Hammers et al., 2019) in isolation, but not both in an integrative manner (but see Cooper et al., 2021). Given that these two components of fitness can follow different ageing trajectories (Jones et al., 2014), and that costs of reproduction, and alleviation by helpers, may be important for survival (Kirkwood, 1977), a full picture of how cooperative breeding can shape age‐specific fitness acquisition requires a detailed understanding of both reproductive and survival components.

Intuitively, the focus of load‐lightening research has been on the effect of the helping behaviour on breeder fitness at different ages. In theory, however, if the costs and benefits associated with helping are (st)age dependent, we may then expect (st)age‐dependent expression and fitness consequences of the helping behaviour from the helper's perspective (McNamara & Houston, 1996; Rodrigues, 2018). There are plentiful examples of such age polyethism of helper behaviour in nature (Field et al., 2006; Zöttl et al., 2016). Workers of the termite *Neocapritermes tracua*, for example, switch helping behaviour from a feeding role to self‐sacrificing colony defence as they age and their feeding efficiency declines (SobotnÍk et al., 2012). Despite this evidence, however, we are not aware of any study that has explicitly tested for senescence in helping behaviour.

**FIGURE 1:**
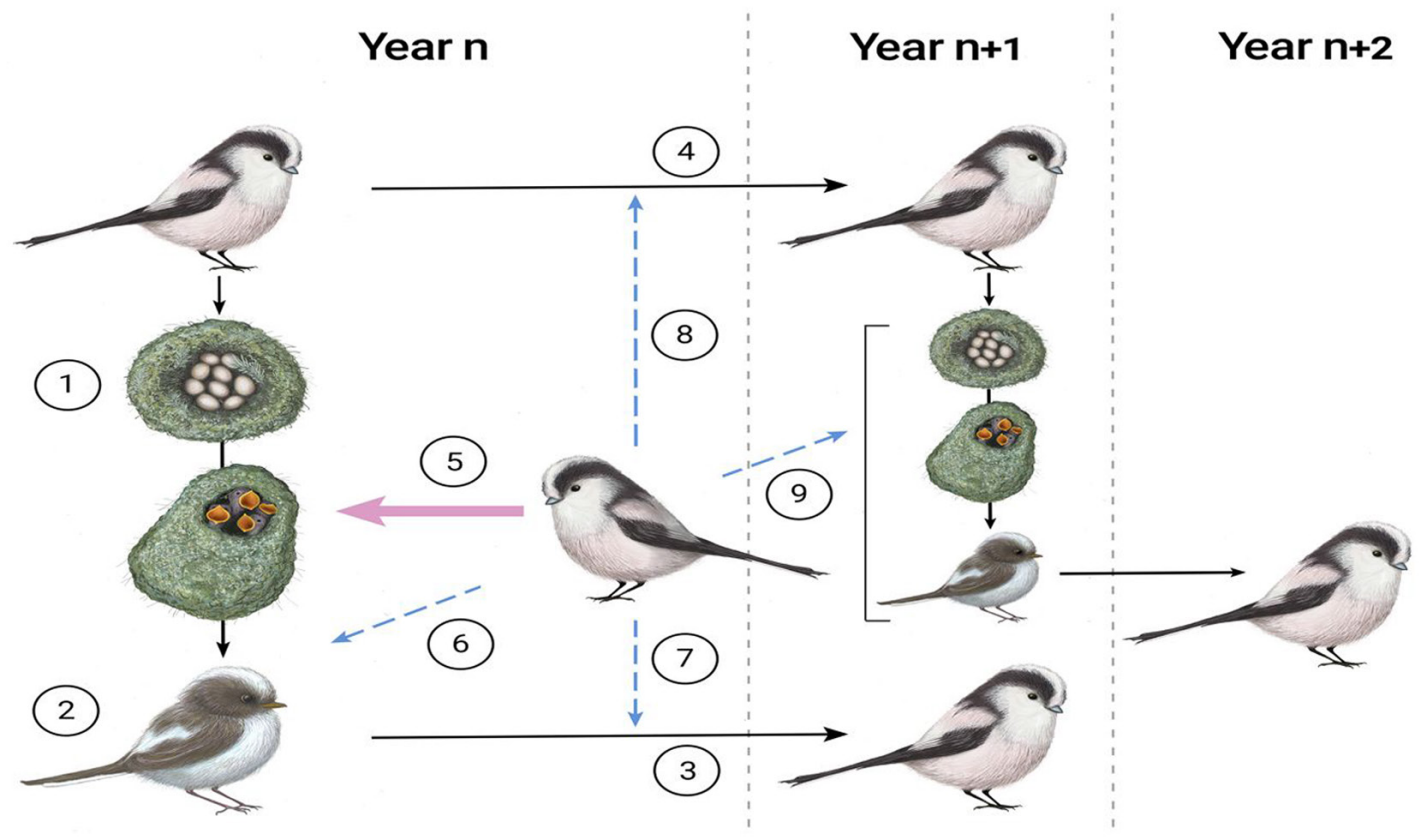
The life cycle of long‐tailed tits *Aegithalos caudatus* and key demographic events. Numbers indicate demographic events we investigated, the models for which are displayed in Table 1. In year *n*, adult long‐tailed tits produce clutches of around 8–12 eggs (1) in socially monogamous pairs which develop via a nestling and fledgling (2) stage ultimately to recruit into the population in year *n* + 1 (3). Adults may also survive (4) to year *n* + 1, with a mean life expectancy of 1.67 years. At the nestling stage, breeders may receive assistance rearing their brood from individuals in the population who have failed to breed—redirected helpers (5). Helpers tend to be related individuals, whose effect is not to increase the number of fledglings at a nest (6), but instead the probability a given fledgling recruits into the population (7). Helpers have been shown to have limited effects on breeder survival (8), while their effects on breeder fitness in year *n* + 1, that is production of recruits in year *n* + 2 (9), is unknown.

Here, we investigate ageing patterns in the long‐tailed tit *Aegithalos caudatus*, a facultative cooperative breeder with redirected helping. In this species, all individuals attempt to breed in every year of their life. If nests fail early in the season, individuals may attempt to breed again, but later in the seasons they may instead choose to help other breeding pairs in raising their offspring by feeding nestlings and fledglings (MacColl & Hatchwell, 2002; Hatchwell, 2016; Figure 1). The helping behaviour is altruistic, favoured by the indirect fitness benefits individuals gain when increasing the productivity of broods to which they are related (Hatchwell et al., 2014). Given that individuals can switch between breeding and helping roles both within and across breeding seasons, it is important to investigate age‐related patterns of both breeding and helping behaviour to fully understand individual ageing patterns in this species (Figure 1; Table 1).

**TABLE 1:** Summary of models investigated in this study. Terms: age at last reproduction (ALR), reproductive effort (RE), presence or absence of helpers (helpers), relative lay date (RLD), relative fledge date (RFD).

	Figure 1	Response variable of demographic events investigated	Fixed effects	Random effects
Ageing patterns of breeder survival and reproduction	1, 2, 3, 4	Clutch size	Age, Age^2^, ALR	Identity, Year
		Fledglings	Age, Age^2^, ALR, Sex	Identity, Breeding Pair Identity, Year
		Direct fitness	Age, Age^2^, ALR, Sex	Identity, Breeding Pair Identity, Year
		Survival	Age, Age^2^, Sex, Direct Fitness, RE	Identity, Year
Effects of helpers on breeders' ageing patterns	6,7,8,9	Current reproduction: fledgling production and recruit production	Age, Age^2^, ALR, Sex, Helpers	Identity, Breeding Pair Identity, Year
		Survival	Age, Age^2^, ALR, Sex, Helpers	Identity, Year
		Future reproduction: RLD, clutch size, fledgling production and direct fitness (all in year *n* + 1)	Age (*n* + 1), Age^2^ (*n* + 1), Helpers (*n*), ALR, Brood size (*n*), Recruits (*n*), RFD (*n*), Spring temp, Winter rainfall	Identity, Year
Ageing patterns of helping behaviour	5, 7	Decision to help	Age, Age^2^, ALR, RLD, RE	Identity, Year
		Probability a fledgling recruit	Helper mean age, fledgling sex, RFD, Helpers (number), relatedness	Identity, Year
		Indirect fitness	Age, Age^2^, ALR, Sex	Identity, Year

This study species provides many benefits for senescence research in social animals. First, given the short adult life spans of individuals (mean = 1.67 years), lifetime reproductive success (LRS) can be straightforwardly quantified for many individuals. Second, as helping is performed only by failed breeders, and there are no direct fitness benefits of helping (Meade & Hatchwell, 2010), we can clearly separate direct and indirect components of inclusive fitness. This distinction allows us to investigate ageing patterns in these components of fitness independently, and then subsequently how helpers may alter age trajectories of components of breeder fitness. Finally, these fitness components can further be broken down into moments of the reproductive schedule for each year of a bird's life. This breakdown scales from clutch sizes through to the number of fledglings, to the number of recruited offspring for breeders, and from the decision to help to the probability that an offspring receiving help successfully recruits into the breeding population. In this species, therefore, we can investigate senescence from the level of inclusive fitness (Hamilton, 1964), and down through multiple fitness components that contribute to this metric, rendering the long‐tailed tit an excellent study system for investigating senescence.

In this study, we undertook a comprehensive analysis of ageing in long‐tailed tits that includes (a) ageing patterns of breeder survival and reproduction [including age‐related changes in extra‐pair paternity (EPP)], (b) the effects of helpers on these patterns and (c) ageing patterns of helper behaviour (see Table 1). First, for breeders, we expected survival and reproduction to follow an increase from maturity to a peak followed by a senescent decline, as is evident in other passerines and in general across animals (e.g. Bouwhuis et al., 2009; Clutton‐Brock, 1984). Given that the long‐tailed tit is a short‐lived species, we expected a trade‐off between reproductive output and survival, which we tested for by including direct fitness and reproductive effort (see Materials and Methods) in models of survival. In our study population, females exhibit a degree of promiscuity, with an estimated 11% of offspring in 30% of nests being the product of extra‐pair mating (Green & Hatchwell, 2018). To fully understand the ageing patterns of reproduction in this species, we therefore also explored the relationship between (a) female age and production of extra‐pair offspring and (b) male age and the probability of both gaining EPP and losing paternity to extra‐pair sires. We had no clear a priori prediction for how EPP should vary with female age, given the diversity of results obtained for other species (Jennions & Petrie, 2000; Raj Pant et al., 2020). In the case of males, for which we have some evidence for variation in quality (Meade & Hatchwell, 2010), we expected a male's life span (which we assume is a predictor of quality) to be positively associated with the probability of gaining EPP and negatively associated with the probability of being cuckolded. Second, we tested for load‐lightening effects of helpers on breeder current reproduction, survival and future reproduction. Previous work in this species has shown that helpers boost long‐term (recruit number) but not short‐term (clutch size and brood size) productivity of breeders (Hatchwell et al., 2004). We therefore expected the presence of helpers to reduce age‐related decline in measures of recruit production but not short‐term productivity. Previous research has also found that helpers boost survival of male breeders caring for large broods, but found no significant effects of being helped in year *n* on an individual's clutch size or timing of breeding in year *n +* 1 (Meade et al., 2010). We extend these analyses to ask whether being helped in year *n* influences the probability of fledging offspring and accruing direct fitness in year *n +* 1. Finally, given that previous work in this species has suggested that helpers are relatively high‐quality individuals (McGowan et al., 2003; Meade et al., 2010), we expected to find limited age‐related decrease in helping performance, measured through (a) indirect fitness accrual and (b) the probability a helped fledgling recruited into the population. If senescence occurs in the study population, however, this may influence an individual's decision to help or not given that helping is a costly behaviour (Hatchwell et al., 2014).

## 2 MATERIALS AND METHODS

### 2.1 Study site and collection of data

Since 1994 (data here up until 2019), a population of long‐tailed tits has been studied within a 2.5 km^2^ study site in the Rivelin Valley, Sheffield, UK (52°23′N, 1°34′W). The animal study was reviewed and approved by the University of Sheffield Ethical Review Committee (Project Applications and Amendments Sub‐Committee). Every year, nestlings are ringed with a unique colour‐ring combination, and blood samples are taken under Home Office Licence (PPL7007834) for genetic analysis. Over 95% of unringed adults that appear in the study population are also colour‐ringed and blood‐sampled; these unringed individuals are assumed to be immigrants born outside the study site. Individuals from whom blood samples are taken are genotyped at 19 microsatellite loci (Adams et al., 2015); sharing of alleles at these loci provides an estimate of pairwise relatedness between individuals using the method of Queller and Goodnight (1989). Body mass (±0.1 g) and tarsus length (±0.1 mm) are recorded for offspring on day 11 after hatching, and upon first capture for new immigrants in the population. Immigrants are assumed to be aged one in the year of their first sighting, based on the observations that individuals tend to move relatively short distances following their first breeding season (Hatchwell, 2016). The probability of resighting individuals in this system is high (92% and 83% for males and females respectively; Gullett et al., 2014), and so individuals are assigned a year of death equivalent to the year in which they were last sighted.

In each breeding season, clutch size, number of fledglings and the presence of helpers were recorded for each nest following the methods of Hatchwell et al. (2004). The following year (*n +* 1), surviving fledglings are recorded as recruits for breeders in year *n*. Recruits were converted to genetic offspring equivalents following Green and Hatchwell (2018) to give measures of direct fitness (for breeders) and indirect fitness (for helpers) that are directly comparable and can be summed to obtain inclusive fitness (Hamilton, 1964). To calculate indirect fitness, we first estimated the average effect of a given number of helpers on the probability of offspring recruitment. Using this information, we then estimated the average fraction of a recruit that is attributable to a helper, for a given number of helpers at a nest. A helper's indirect fitness was then obtained by multiplying this fraction by the relatedness between the helper and any offspring that successfully recruited. Where multiple offspring recruited, the average relatedness between these and the helper was calculated. Helpers received no indirect fitness if no recruits were produced from the nest they helped, nor if their relatedness to recruits was ≤ 0. The direct fitness of an individual in a given year of their life was the number of recruits produced in a year minus the fraction of recruits attributable to any helpers. The remaining fraction was then halved twice, first to account for the contribution of the other breeding partner to the recruit produced, and second to account for the average relatedness of 0.5 between parents and offspring. The inclusive fitness accrued by a bird in a year of their life is simply the sum of the indirect and direct fitness they accrue in the year. Note that the measures of fitness we calculated correct for parentage lost and gained through extra‐pair matings. Fitness metrics were calculated for a total of 813 individuals, for which complete life‐history information was available.

We also used information on an individual's total reproductive investment across all nesting attempts in a given year to quantify a measure of reproductive effort (RE), following Tarwater and Arcese (2017). In long‐tailed tits, incubation is performed exclusively by females, who are fed by their partners, while provisioning of nestlings is carried out by both sexes at approximately equal rates (Hatchwell, 2016). For females (*n* = 339), we calculated RE as the sum of an incubating (number of eggs × days incubating) and a provisioning (number of chicks × days provisioning) term. For example, an individual that lays 10 eggs, incubates for 13 days and then provisions these 10 chicks for 13 days would have a reproductive effort of 260. For males (*n* = 287), RE was calculated as the number of days their breeding partner spent incubating plus the same provisioning term used for females.

### 2.2 Analyses of age‐related components of fitness

Linear and quadratic effects of age were included in mixed‐effects models to test for age‐related patterns in the different components of fitness (Table 1). A significant quadratic effect of age may be due to improvement early in life rather than senescence, however, and therefore we performed ‘post‐peak analysis’ to test for a significant negative linear relationship between age and the respective fitness component. This methodology excludes the quadratic effect in analyses of individuals of peak age and older with respect to a particular fitness component (Bouwhuis et al., 2009), where peak age represents the age with the highest mean value of relevant fitness components. A significant negative term of linear age in such an analysis would, if present, provide more reliable evidence of senescence.

When investigating population‐level patterns of ageing, it is imperative to control for changes in the phenotypic composition of the population due to between‐individual variation in quality (Van de Pol & Verhulst, 2006; Vaupel & Yashin, 1985). Age at last reproduction (ALR) was therefore included in all models testing for reproductive senescence to control for selective disappearance of ‘poor‐quality’ individuals (Van de Pol & Verhulst, 2006; Table 1). Long‐tailed tits always attempt to reproduce in their first year (Hatchwell, 2016), and so there is no need to control for selective appearance in our study population. In addition, we included the year (1994–2019) in which the data were collected and individual identity as random effects (Table 1). This step allowed us to account for both environmental variation and non‐independence caused by repeated sampling of individuals across years. For models of reproductive senescence in which the two sexes were analysed together, we included a Pair ID term (mother ID plus social father ID) to control for non‐independence caused by contributions of both parents to the production of fledglings and recruits.

Low sample sizes at late age classes can be an issue in detecting senescence (Nussey et al., 2008). Therefore, we reran our models with birds aged 4 and older classified as 4+ to boost sampling power. Also, through preliminary analysis, we identified that one bird (ID 1978) had a particularly successful year in its sixth year of life (inclusive fitness = 0.61, mean across population for a single year = 0.06) and so we reran our models with this bird excluded from the dataset. Throughout the results section, we report results of these alternative analyses only where they generate qualitatively different results. All analyses were conducted in R (R Core Development Team, 2015) and in RStudio (v 4.0.2). Mixed‐effects models were run using the lme4 package (Bates et al., 2015). Finally, access to the study site was heavily restricted in 2001 following an outbreak of foot‐and‐mouth disease, resulting in uncertainty about (a) recruitment of offspring born in 2000 and (b) the survival of adult birds last observed in 2000. Thus, we removed these data from survival and direct fitness analyses.

#### 2.2.1 Ageing patterns of breeder survival and reproduction

##### Reproduction

We modelled clutch size with a Poisson error structure and for females only, given they are likely to have principal control over the number of eggs laid. For individuals that laid multiple clutches in the same year, we chose to analyse the size of the first clutch only to standardise measures across individuals and reflect condition at the start of the breeding season. As a result of the high level of whole brood mortality in long‐tailed tits (>70%; Hatchwell, 2016), the number of fledglings produced and the amount of direct fitness accrued are heavily zero‐inflated, so we modelled them with a compound Poisson error structure. Extra‐pair paternity is corrected for in these analyses through paternity assignment (see Supplementary Information). We modelled both sexes together including sex as a covariate and a Pair ID term to account for non‐independence. We also tested for an interaction between sex and age to test for sex‐specific age trajectories of reproduction. For reproductive components of fitness, we were also able to account for terminal effects (see Supplementary Information; Clutton‐Brock, 1984; Coulson & Fairweather, 2001). Finally, we used biometric information (see Supplementary Information) to test for correlations between lifetime inclusive fitness and life span with body mass.

To test for ageing patterns of extra‐pair paternity, we investigated females and males separately using information from the genetic pedigree of the study population (see Supplementary Information). For females (*n* = 225), we modelled the probability they produced offspring from multiple males in a given year of their life as a function of their age, ALR and the difference in age between them and their social male partner using a binomial error distribution. The rates of conspecific brood parasitism in long‐tailed tits are negligible (Green & Hatchwell, 2018; Hatchwell et al., 2002), so the social mother was assumed to have maternity in all cases. For social fathers (*n* = 213), we first modelled the probability they raised broods including at least one extra‐pair offspring using the same fixed effects. In both models, we also included the number of nestlings as a fixed effect to control for the increased likelihood of extra‐pair paternity in larger groups (Raj Pant et al., 2019). Of the 429 males for which we had accurate lifetime recruit data, 46 were known to have sired at least one extra‐pair chick. We modelled the probability males gained EPP with age (linear and quadratic) and ALR as fixed effects, and individual identity as a random effect. Due to the small number of males gaining EPP, attempts to account for Year as a random effect led to the model failing to converge.

##### Survival

We used the probability of individual survival from 1 year to the next to investigate actuarial senescence with a binomial error distribution. We included age (linear and quadratic) and sex as fixed effects, and an interaction between the two to test for differences in age trajectories of survival between the sexes. We also included the mean daily rainfall (mm) from 1 October in year *n* to 1 March in year *n +* 1, previously shown to have a negative effect on survival in long‐tailed tits (Meade et al., 2010). Weather data were provided by Weston Park Weather Station, Museums Sheffield, 5 km from the Rivelin Valley study site. Finally, we included direct fitness in year *n* as a further fixed effect to test for trade‐offs between current reproduction and future survival. We performed an additional survival model for individuals that we had RE data for including both RE and direct fitness as fixed effects.

#### 2.2.2 Effects of helpers on breeders' ageing patterns

As helpers only help at nests that have survived beyond the incubation period, they are inevitably associated with more productive nests (Hatchwell, 2016). Helpers can arrive at any point between hatching and the fledgling stage and therefore, when testing for an effect of helpers on breeder fitness, we restricted our analyses to just those nests that produced fledglings and therefore could have received help, resulting in 249 breeding attempts for 390 individuals (199 females and 191 males).

##### Current reproduction

To test for the effect of helpers on the productivity of successful breeders, fledgling number was modelled with a Poisson error structure. Since our fitness metrics account for the contribution of helpers to recruit production, we model the effect of helpers on absolute recruit number rather than direct fitness with a negative binomial error structure. We included interactions between breeder age (linear and quadratic) and whether helpers were present or not at their nest to test for differences in age trajectories of reproduction between breeders that did and did not receive help.

##### Survival

To test for the effects of helpers on breeder survival, we first attempted to recapture the results of Meade et al. (2010) with 10 more years of data. We therefore included the breeder's brood size, relative fledge date and whether they produced recruits or not as fixed effects, alongside breeder age (linear and quadratic) and whether helpers were present or not. In their analysis, Meade et al. (2010) found that helping confers survival benefits to breeders, but that this effect was restricted to male breeders with large broods. We therefore fitted pairwise interactions between helping and sex and brood size to test whether survival benefits of help depended on breeder sex and brood size. Finally, we also included the mean daily rainfall (mm) from 1 October in year *n* to 1 March in year *n* + 1, as this has previously been shown to have a negative effect on the survival of females (Meade et al., 2010).

##### Future reproduction

We extended the work of Meade et al. (2010) to investigate helper effects on future reproduction of breeders. For individuals that produced fledglings in year *n* and therefore could have received help, we tested for an effect of whether they received help or not on clutch sizes and relative lay dates in year *n* + 1. We then tested whether being helped in year *n* influenced the probability of fledging offspring or accruing direct fitness through recruit production in year *n* + 1. Fixed effects included whether the individual was helped, their brood size, and whether they produced recruits, in year *n*, and their age (linear and quadratic) in year *n* + 1. We also included the mean daily rainfall throughout winter and the mean minimum daily temperature in degrees from 15 February to 15 March in year *n* + 1 (spring temperature) as further fixed effects.

#### 2.2.3 Ageing patterns of helper behaviour

To investigate senescence of helping behaviour, we first tested for age patterns of indirect fitness accrual across the population. We then modelled the probability a fledgling recruited into the population as a function of its helpers' mean age (764 fledglings across 99 nests between 1995 and 2018). We modelled success or failure of recruitment by each fledgling using a binomial error distribution. We included relative fledge date (date of fledging relative to the 1 May), the number of helpers and the sex of the fledgling (to account for female‐biased dispersal) as fixed effects (Green & Hatchwell, 2018). We also modelled the mean relatedness of the fledgling to its helpers as a fixed effect, given helpers have been shown to adjust their provisioning rates in accordance with their relatedness to nestlings (Leedale et al., 2018; Leedale et al., 2020; Nam et al., 2010).

We also tested whether the age of an individual influenced the decision to help. To do so, we restricted our analysis to birds that had the opportunity to help (98 helping decisions by 90 females and 163 helping decisions by 144 males). For this analysis, we only included birds that had failed to fledge offspring in a particular year and had at least one viable helping option (i.e. a nest of at least one first‐order relative containing nestlings) in the study site on the day that its final nesting attempt failed. We also restricted the analysis to individuals for which we had information on RE from failed breeding attempts in the year in question. In these analyses, fixed effects were thus age, ALR, RE and relative lay date.

## 3 RESULTS

### 3.1 Ageing patterns of breeder reproduction and survival

#### 3.1.1 Reproduction

**FIGURE 2:**
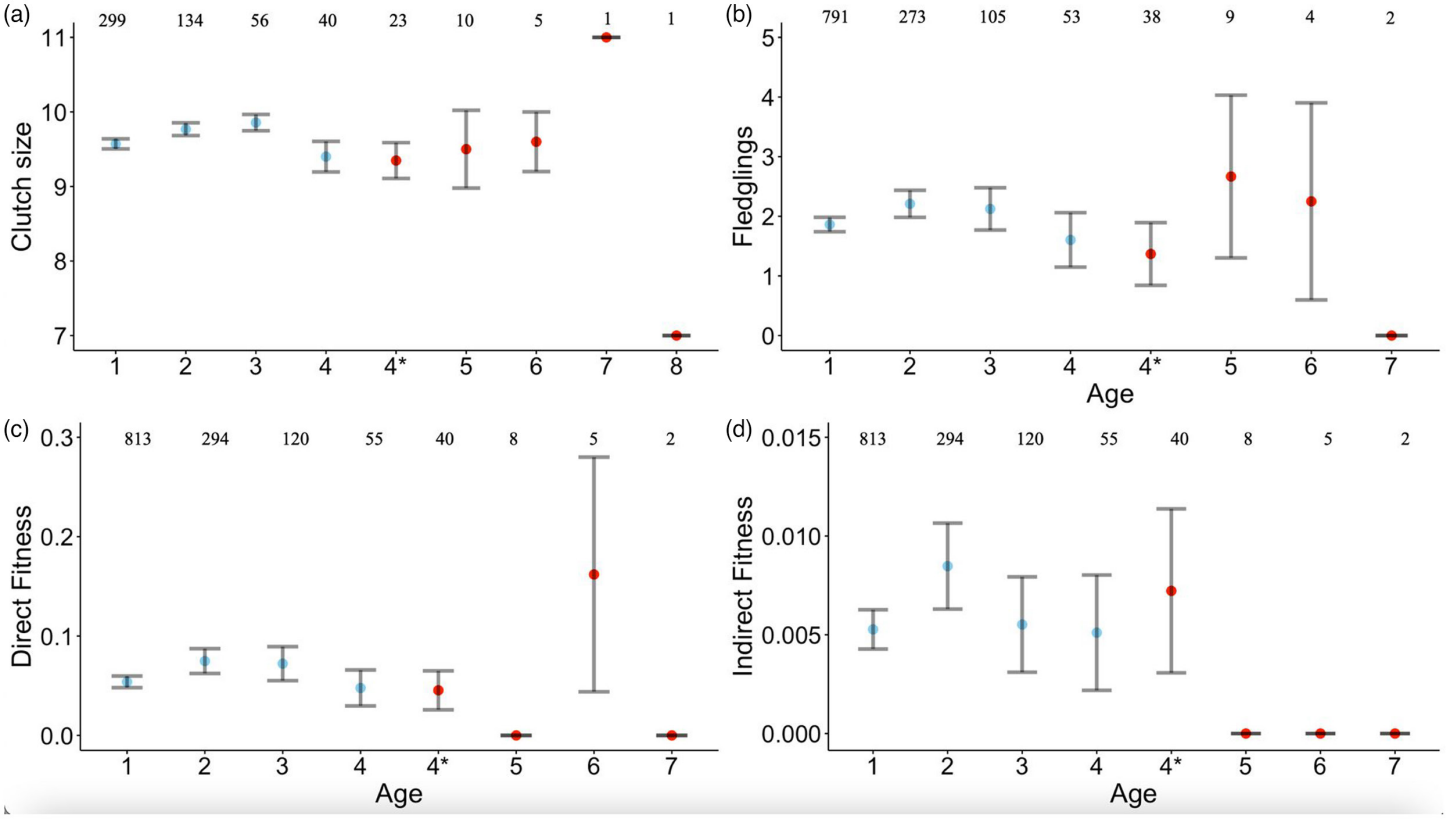
Age patterns of reproduction. Mean ± *SE* (a) clutch sizes of females, (b) number of fledglings, (c) direct fitness and (d) indirect fitness in relation to individual age. Results are presented both for when individuals aged 4 and older are grouped as 4+ (blue), and as the raw means for age classes 4 to 7/8 (red). Sample sizes for each age class are displayed above each data point.

**FIGURE 3:**
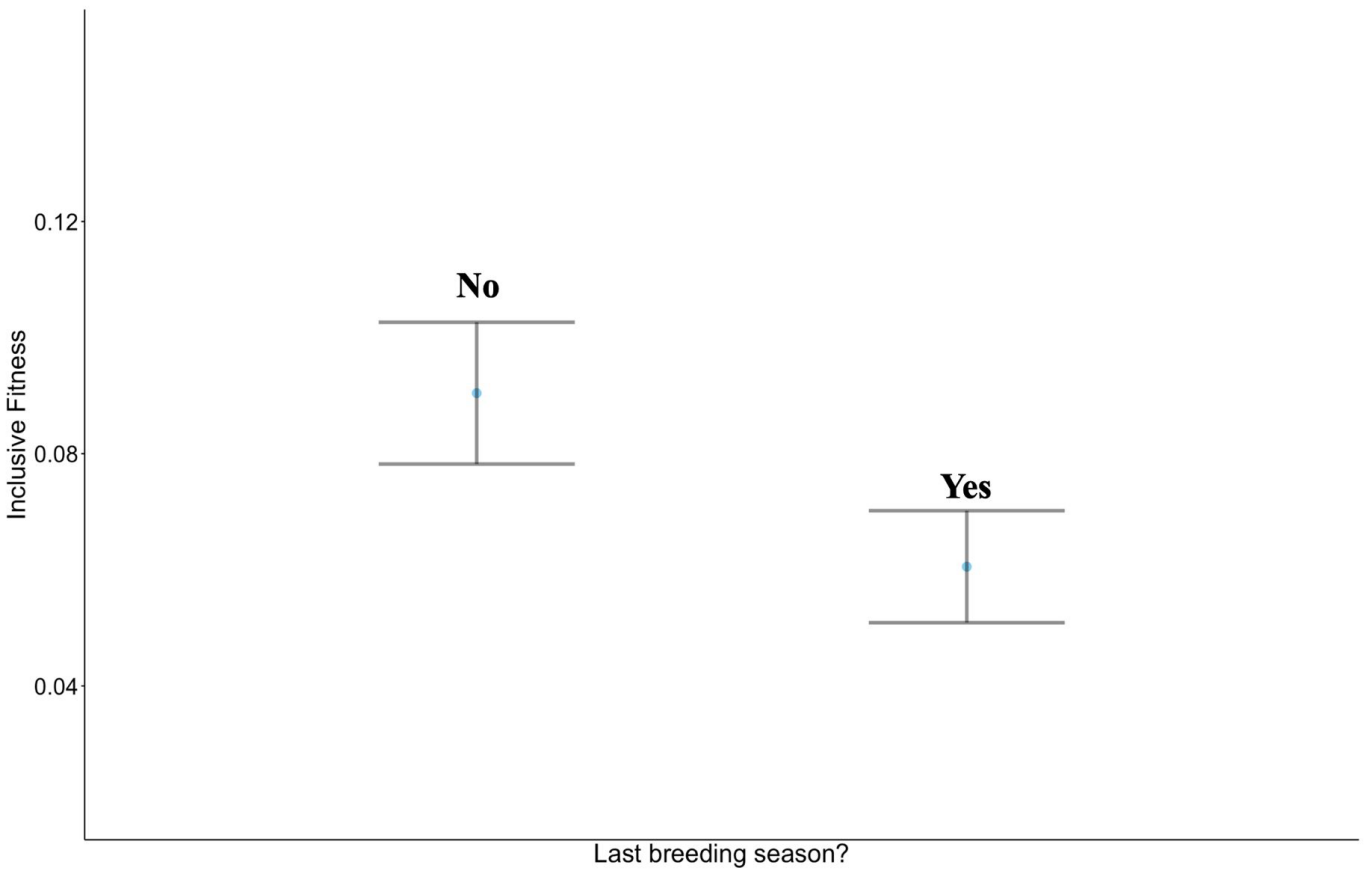
Inclusive fitness in an individual's last breeding season. Mean ± *SE* inclusive fitness for individuals in breeding seasons throughout their life span (No) compared to their final breeding season (Yes).

We found variable relationships between age and different moments of the reproductive schedule. First, we found no relationship between maternal age and clutch size (*χ* ^2^ = 0.76, *p* = 0.38; Figure 2a; Table S1a). Analysing both sexes together, we found a significant quadratic relationship between breeder age and number of fledglings produced when older individuals were clustered as 4+ and individual 1978 was removed (*χ* ^2^ = 4.28, *p* = 0.04; Figure 2b; Table S1b). Post‐peak analysis revealed this was likely due to early life improvement, with no significant negative linear effect of age on fledgling production for individuals older than 2 (Age 3+: *β* = −0.20 ± 0.20, *χ* ^2^ = 1.04, *p* = 0.31). A similar quadratic effect was observed for direct fitness (*χ* ^2^ = 3.90, *p* = 0.05; Figure 2c; Table S1c) and inclusive fitness (*χ* ^2^ = 4.28, *p* = 0.04; Figure S1; Table S1d). These were again due to early life improvements, with no significant negative linear effects of age in post‐peak analyses (direct fitness: Age 2+: *β* = −0.37 ± 0.27, *χ* ^2^ = 2.29, *p* = 0.13; inclusive fitness: Age 2+: *β* = −0.28 ± 0.25, *χ* ^2^ = 1.29, *p* = 0.25). However, we found that individuals gained significantly less fitness in their last year of life. In the case of direct fitness, this effect was marginally significant when individuals were grouped as 4+ (*β* = −0.53 ± 0.30, *χ* ^2^ = 3.78, *p* = 0.05) but not when assigned their true age (Table S1c). For inclusive fitness, a significant terminal effect was observed even when individuals were assigned their true age (*β* = −0.55 ± 0.27, *χ* ^2^ = 4.59, *p* = 0.03; Figure 3; Table S1d).

In addition to these age effects, we found significant effects of ALR on fledgling production (*χ* ^2^ = 4.40, *p* = 0.04) and accrual of direct (*χ* ^2^ = 4.36, *p* = 0.04) and inclusive fitness (*χ* ^2^ = 10.93, *p* < 0.001; Table S1b–d), suggesting selective disappearance of poorer quality individuals. ALR was not correlated with nestling mass among philopatric individuals or with adult body mass among immigrants (*p* > 0.30); however, among immigrants, adult body mass was significantly positively correlated with lifetime inclusive fitness (Figure S2; *β* = 0.42 ± 0.19, *χ* ^2^ = 12.36 *p* = 0.001). For philopatric recruits, we found no evidence that receiving help as a nestling had a significant impact on life span or lifetime accrual of inclusive fitness (*β* = 0.45 ± 0.29, *χ* ^2^ = 2.42 *p* = 0.12). Finally, we found no evidence of age effects on a female's propensity to produce extra‐pair offspring, nor a male's likelihood of gaining, or losing out to, EPP (Table 2). Furthermore, there were no effects of ALR in either the male or female analysis, indicating that birds with different reproductive life spans did not have significantly different rates of extra‐pair paternity.

**TABLE 2:** Models investigating the effect of age on extra pair paternity (EPP) in females and males. For females, we modelled the probability that a female sired at least one nestling that was not the genetic offspring of the social father. For males, we modelled the probability that the social father was cuckolded. Age at last reproduction (ALR) and brood size (*N*) were also included as fixed effects. Year and bird IDs were included as random effects.

Fixed effects	*β*	*SE*	*χ* ^2^	*p*
Females				
Age	0.65	0.55	1.47	0.22
Age^2^	−0.13	0.10	1.82	0.18
ALR	0.004	0.14	0.001	0.97
*N*	0.002	0.06	0.001	0.97
Males: probability of losing EPP				
Age	−0.43	0.45	1.02	0.31
Age^2^	0.10	0.07	2.26	0.13
ALR	−0.07	0.14	0.24	0.63
*N*	0.01	0.07	0.03	0.86
Males: probability of gaining EPP				
Age	−0.90	1.03	0.85	0.36
Age^2^	0.24	0.20	1.90	0.17
ALR	−0.09	0.52	0.03	0.86

*Note*: Models were fitted with a binomial error structure.

#### 3.1.2 Survival

**FIGURE 4:**
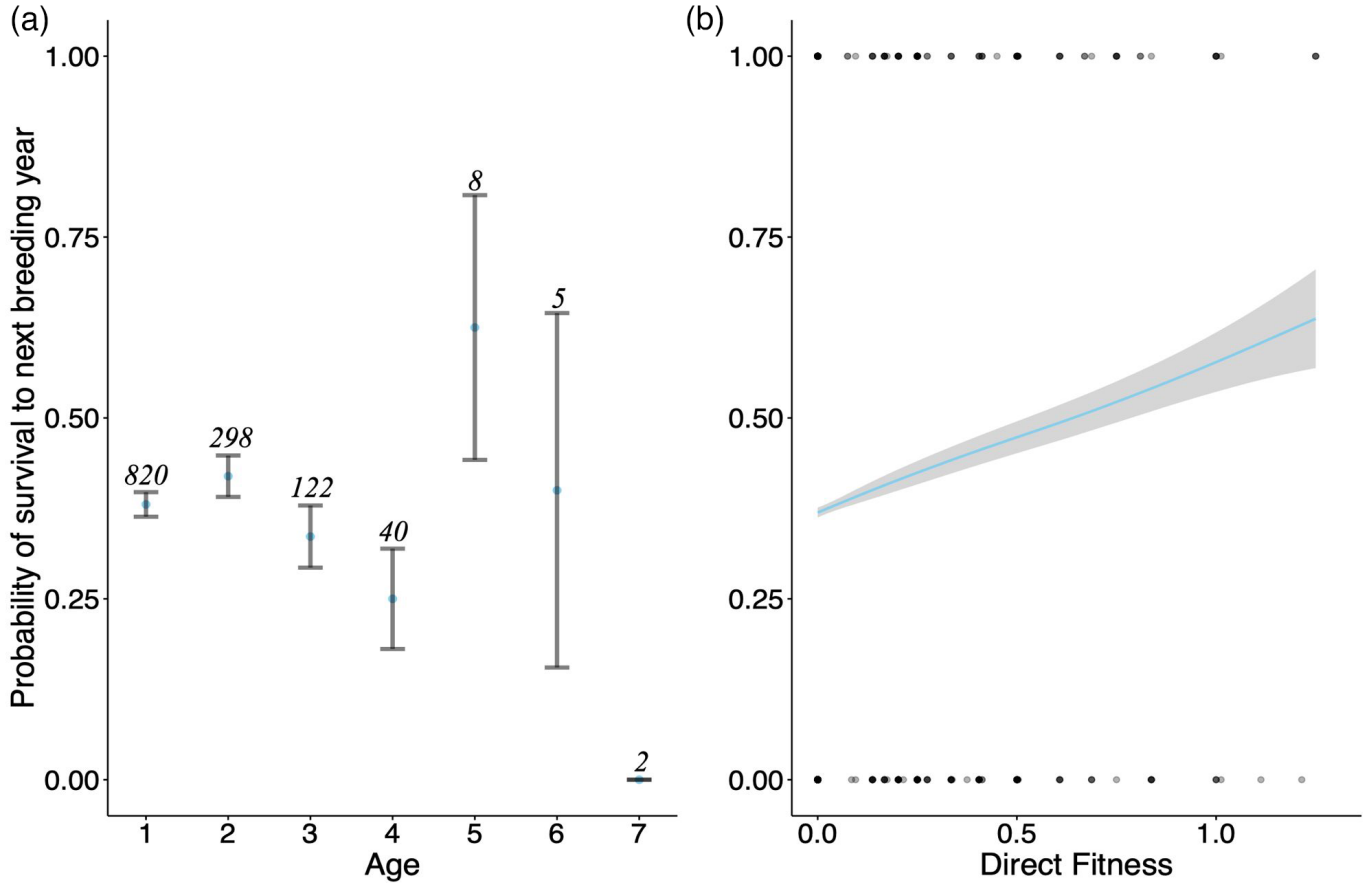
Age patterns of survival. (a) Mean ± *SE* probability of adult survival from one breeding season to the next with respect to age. (b) The predicted probability ±*SE* of survival to the next breeding season as a function of an individual's direct fitness in the current breeding season.

Our overall mean rate of survival across the population was lower (0.38) than previously recorded (0.50; Hatchwell, 2016). However, this is likely an artefact of our study sample of individuals. The individuals included in our survival analyses were only those for which we could calculate accurate values of fitness across their whole life span. This therefore biases our sample to birds for which no breeding seasons were missed, and in particular birds observed breeding for a single year (i.e. shorter lived birds). There was no significant effect of age on the probability of adult survival across the whole population (*χ* ^2^ = 0.05, *p* = 0.81; Figure 4a; mean survival between years = 0.38), and no difference between the sexes in their age trajectories of survival (Age × Sex: *χ* ^2^ = 0.34, *p* = 0.56; Age^2^ × Sex: *χ* ^2^ = 0.08, *p* = 0.78). We found no significant effect of RE on the probability of survival for females (*χ* ^2^ = 0.60, *p* = 0.44) or males (*χ* ^2^ = 0.23, *p* = 0.63; Table S1f). Rather, individuals that gained direct fitness in a given year through the production of offspring that successfully fledged and recruited were then more likely to survive to the following year (*β* = 0.78 ± 0.34, *χ* ^2^ = 5.51, *p* = 0.02; Figure 4b). When included in models testing for the effects of RE, there was a significant effect of direct fitness on the survival of females (*β* = 1.45 ± 0.51, *χ* ^2^ = 8.56, *p* = 0.003) but not males (*χ* ^2^ = 0.03, *p* = 0.87; Table S1f). Winter rainfall was also negatively associated with female (−0.73 ± 0.28, *χ* ^2^ = 5.54, *p* = 0.02) but not male survival (*χ* ^2^ = 0.48, *p* = 0.49; Table S1f).

### 3.2 Effects of helpers on breeders' ageing patterns

In line with previous work, we found no effect of helping on fledgling production (*β* = 0.007 ± 0.04, *χ* ^2^ = 0.04, *p* = 0.85) but a significant positive effect of the number of helpers on the probability that fledglings recruited into the breeding population (*β* = 0.16 ± 0.06, *χ* ^2^ = 7.02, *p* = 0.01). Greater breeder survival to year *n* + 1 was not predicted by the presence of helpers for males (*β* = −0.46 ± 0.28, *χ* ^2^ = 2.83, *p* = 0.09), nor females (*β* = −0.10 ± 0.27, *χ* ^2^ = 0.14, *p* = 0.71). Instead, for males, higher survival to year *n* + 1 was associated with a later relative fledge date of offspring in year *n* (*β* = 0.04 ± 0.02, *χ* ^2^ = 6.24, *p* = 0.01), while for females, survival to the following breeding season was negatively related to winter rainfall levels (*β* = −1.01 ± 0.29, *χ* ^2^ = 11.15, *p* = 0.001) and positively related to the number of offspring surviving to recruit (*β* = 0.69 ± 0.28, *χ* ^2^ = 5.95, *p* = 0.01). We found no significant interactions between helper presence and age (linear nor quadratic) for fledgling production (Age × Helpers: *χ* ^2^ = 0.08, *p* = 0.77; Age^2^ × Helpers: *χ* ^2^ = 0.01, *p* = 0.92) or recruit production (Age × Helpers: *χ* ^2^ = 0.04, *p* = 0.85; Age^2^ × Helpers: *χ* ^2^ = 0.08, *p* = 0.77) nor survival (Age × Helpers: *χ* ^2^ = 0.59, *p* = 0.44; Age^2^ × Helpers: *χ* ^2^ = 0.60, *p* = 0.44). Thus, although helpers boost breeders' production of recruits, they appear to do so in an age‐independent manner and do not alter the slope of the age trajectories of breeder fitness. Finally, there were no effects of helpers on breeder future reproduction: receiving help in year *n* did not influence clutch size, relative lay date, the probability of fledgling offspring or the probability of accruing direct fitness in year *n +* 1 (all *p* > 0.57; Table S2).

### 3.3 Ageing patterns of helper behaviour

There was no significant effect of age on the amount of indirect fitness accrued by helpers (Figure 2d), but birds with longer life spans were likely to gain more indirect fitness (Table 3). After finding a positive effect of direct fitness on breeder survival, we tested whether greater nest productivity conferred survival benefits to helpers as well. At the nests where helpers provided help, however, there were no effects of the number of fledglings (*n* = 159: *β* = −0.02 ± 0.10, *χ* ^2^ = 0.05, *p* = 0.83), nor the number of recruits (*n* = 161: *β* = 0.11 ± 0.11, *χ* ^2^ = 0.97, *p* = 0.32) on the probability that a helper survived to the next year. These results reaffirm the lack of direct fitness benefits of helping in this system (Meade & Hatchwell, 2010).

**TABLE 3:** Models investigating the effect of age on helper behaviour. We investigated the effect of age on (i) indirect fitness accrued, (ii) the probability a helped fledgling recruit into the population and (iii) the decision to engage in helping behaviour. See Supplementary Information for modelling of Terminal Investment (TI).

Fixed effects	*β*	*SE*	*χ* ^2^	*p*
Indirect fitness				
Age	0.37	0.72	0.36	0.55
Age^2^	−0.14	0.16	1.19	0.28
Sex	2.49	0.80	20.86	**<0.001**
ALR	0.50	0.26	5.18	**0.02**
TI	−0.38	0.63	0.56	0.46
Prob. fledgling recruits				
Helper age^a^	0.11	0.11	0.94	0.33
Sex of fledgling	0.87	0.21	18.07	**<0.001**
Relative fledge date	−0.01	0.02	0.60	0.44
Number of helpers	0.41	0.20	4.23	**0.04**
Relatedness of fledglings to helpers^a^	1.60	0.78	4.28	**0.04**
Decision to help by males				
Age	−0.87	0.84	1.14	0.29
Age^2^	0.12	0.18	0.51	0.47
Relative lay date	−0.02	0.03	0.36	0.55
Reproductive effort	0.10	0.20	0.24	0.62
ALR	0.54	0.20	9.08	***0.002***
Decision to help by females				
Age	−1.79	1.44	1.40	0.24
Age^2^	0.44	0.28	2.23	0.14
Relative lay date	−0.05	0.06	0.72	0.40
Reproductive effort	0.78	0.35	5.55	**0.02**
ALR	−0.29	0.31	0.96	0.33

*Note*: The indirect fitness model was fitted with a compound Poisson error structure, while the other two models were fitted with a binomial error structure. Significant (*p* < 0.05) terms are given in bold.

^a^ Where breeders were assisted by multiple helpers, average values across all helpers were calculated.

There was no effect of mean helper age on the probability that a fledgling at a helped nest recruited into the breeding population (Table 3). Greater recruitment was detected among males, due to female‐biased dispersal in this study population (Hatchwell, 2016). Furthermore, a higher mean relatedness between a fledgling and its helpers significantly increased the fledgling's probability of recruitment (Table 3). This result is consistent with the finding that helpers increase their provisioning efforts when they have higher relatedness to a brood (Leedale et al., 2018; Leedale et al., 2020; Nam et al., 2010).

Finally, there were no effects of age on an individual's decision to help or not (Table 3). RE was significantly positively associated with the decision to help in females, that is individuals with higher RE in a breeding season were more likely to help (*β* = 0.78 ± 0.35, *χ* ^2^ = 5.55, *p* = 0.02). We did not find this effect in males, but were unable to test for a difference between the slopes of RE on the decision to help or not, since RE was calculated differently for the two sexes. In males, but not females, ALR had a significant positive effect on the decision to help (Table 3), indicating that birds with longer life spans were more likely to help in a given year of their life.

## 4 DISCUSSION

In this study, we investigated age patterns of fitness in a wild population of long‐tailed tits *Aegithalos caudatus*. We found evidence of increased reproductive performance with age from maturity, but a lack of evidence for significant senescent decline. However, breeders gained significantly less fitness in their final year of life. We detected no significant effects of age on survival, which was instead positively correlated with the amount of direct fitness accrued. Helpers had no impact on clutch or brood size, but did have a positive effect on recruit production for breeders in the year that they received help. This effect of helpers on breeder reproduction was (breeder) age‐independent, and, furthermore, helper presence did not affect breeder survival. Considering ageing patterns of helpers themselves, we found no effects of age on (a) the decision to help, (b) the probability that a helped fledgling would recruit and (c) the indirect fitness they accrue. Long‐tailed tits are a short‐lived, small passerine species, threatened by a high rate of extrinsic mortality. The individuals who manage to live substantially longer than average appear to be of higher quality, and our results reflect that this variation in individual quality across the population is of paramount importance for all aspects of this cooperative breeding bird's life history.

### 4.1 Breeder ageing patterns and effects of helpers

The extent to which alloparental care can influence the patterns of senescence, and how age in general may contribute to explaining variation in social behaviour remain key questions in evolutionary ecology (Berger et al., 2018). Across species, the effect of helpers on different components of breeder fitness are variable (positive: Khan & Walters, 2002; Berger et al., 2018; Hammers et al., 2019; negative: Brouwer et al., 2006; no effect: Sharp & Clutton‐Brock, 2010). Long‐tailed tits exhibit an unusual helping system relative to most other cooperative breeders, in which individuals may redirect care to related broods after failing to breed themselves. In this study, helpers were found to boost breeder productivity in terms of number of recruits, in line with previous work (Hatchwell et al., 2014), but had no effect on fledgling number or breeder survival. When comparing nests that received and did not receive help there were no significant differences between the slopes of age trajectories of recruit production. This suggests that although helpers boost the magnitude of breeder recruit production, the effect is independent of breeder age. While previous work on this system had suggested that male breeders benefited from the presence of helpers when raising large broods (Meade et al., 2010), such an effect was not evident in our analysis, which included an additional 11 years of data (Figure S3). Indirect fitness returns from helping thus appear to be generated primarily through the enhancement of offspring recruitment in this species. This conclusion is consistent with the negligible contribution (2.5%) that load‐lightening makes to the indirect fitness benefits that helpers receive from their cooperative behaviour (Hatchwell et al., 2014).

We found consistent evidence of increasing breeder reproductive performance in early life. This is a common pattern among animals (Bouwhuis et al., 2009; Clutton‐Brock, 1984; Sharp & Clutton‐Brock, 2010) that is typically attributed to differential mortality with regard to individual quality (Balbontin et al., 2007; Reid et al., 2003). In our results, this was supported by significant effects of ALR on fledgling production, and direct and indirect fitness accrual, suggesting selective disappearance of poor‐quality individuals. We did not, however, find any effects of age or ALR on the probability of a female engaging in extra‐pair mating, or a male gaining or losing offspring to EPP, adding to the variable results for age effects on EPP among passerines (Cooper et al., 2021; Hsu et al., 2017; Raj Pant et al., 2020). The increase in breeder reproductive performance we found was not followed by significant late‐life decline, which can be difficult to detect in wild populations due to low sampling of older age classes (Nussey et al., 2013). We addressed this issue by using a longitudinal dataset spanning 25 years of individual‐level data and by grouping individuals aged 4 and older into a 4+ category. The quadratic relationships for fitness components (Figure 2) suggest a senescent pattern, but in all components of reproduction, the mean estimate for individuals aged 4 is lower than those of age 4+, meaning that the oldest individuals in the population are driving up the average. We suggest therefore that the absence of significant quadratic effects may be explained by the high performance of the longest‐lived individuals masking weak senescence in both reproduction and survival, rather than by a complete absence of senescence in long‐tailed tits. Although there are growing theoretical (Baudisch, 2011) and empirical (Péron et al., 2019) arguments that senescence and life span are independent, it may be more difficult, statistically, to detect senescence in species that are short lived where estimates of survival and reproductive performance are based on annual measures. Reproduction in long‐tailed tits is annual, but more fine‐grained (e.g. seasonal) estimates of survival within years, combined with other proxies of senescence such as body condition, might provide greater power for detecting senescence in this and other short‐lived species.

### 4.2 Variation in individual quality

In long‐tailed tits, ALR is consistently associated with higher fitness (Table 1). Evidence of differences in reproductive life spans among individuals determining age‐specific fitness trajectories is widespread across species, from fishes (Suzuki et al., 2010) to baboons (McLean et al., 2019). As we have done here, studies usually consider ALR a reasonable indicator of individual quality, but ALR itself does not describe a causal mechanism that determines ‘quality’. In reality, ‘quality’ will be determined from a plethora of variables, including developmental conditions, body mass, acquisition of resources, luck and better nest locations with reduced risks of predation (Lim et al., 2014). Can we delve deeper into what determines such quality?

In birds, metrics such as relative lay date (Verhulst & Nilsson, 2008) and body mass may offer finer resolution indices of individual quality. Indeed, Meade and Hatchwell (2010) reported that among those long‐tailed tits that failed to breed successfully and had relatives available to help, helpers had earlier relative lay dates compared to non‐helpers. Body mass is known to correlate with age‐specific reproduction and survival both within and across species (e.g. Péron et al., 2019), and for long‐tailed tits is important in determining hierarchies in non‐breeding winter flocks (Napper et al., 2013). We found no evidence that body mass at maturity (immigrant breeders) or as a nestling (philopatric breeders) was associated with life span (ALR), but immigrant body mass was associated with higher lifetime inclusive fitness (Figure S2). Furthermore, some variation in individual ‘quality’ may not be attributable to morphological traits, but may instead be determined by environmental factors. Long‐tailed tits are not territorial (Hatchwell, 2016), but spatial autocorrelation of reproductive performance (e.g. Marrot et al., 2015) has not yet been investigated. Individuals that are able to survive a year may gain information on what particular areas are likely to give them the best chance at successful reproduction.

### 4.3 Positive covariation of individual reproduction and survival

Rather than an expected trade‐off between survival and reproduction, we instead found a positive correlation between breeder reproduction and survival throughout this study. The most likely explanation for the apparent absence of trade‐off between survival and reproduction is differences among individuals in both resource allocation and acquisition (Van Noordwijk & De Jong, 1986). Life‐history trade‐offs between current reproduction and future survival and reproduction are often revealed only when experiments are used to break the link between investment in different functions, caused by these individual quality effects (Zhang & Hood, 2016). There are other, non‐mutually exclusive, explanations for why survival and reproduction in long‐tailed tits may positively covary. As a corollary to an individual's direct fitness accrual being significantly correlated with their chance of survival, we also found that individuals suffered significant decreases in fitness in their final year of life (Figure 1d). This ‘terminal decline’ (Coulson & Fairweather, 2001) could be due to rapid physiological change, with individuals of different quality senescing at varying rates. Countering this, however, we found no evidence of terminal effect in fledgling production (Table S1), though we cannot rule out the possibility that other indicators of fledgling quality, such as body mass, may reflect parental terminal decline.

An alternative explanation, however, could be that higher rates of reproduction really do boost breeder survival through social benefits of producing offspring. In red‐cockaded woodpeckers *Picoides borealis*, each juvenile reduces breeder mortality rate by 16% for males and 26% for females after accounting for helping and territory quality (Khan & Walters, 2002; Walters & Garcia, 2016). This finding was attributed to larger group sizes providing better protection against predation (Walters & Garcia, 2016). Unlike red‐cockaded woodpeckers, long‐tailed tits are not territorial and live in large flocks with relatively fluid membership outside the short breeding season. These non‐breeding flocks are typically composed of all birds associated with a successful nest (breeders, offspring and any helpers), augmented by failed breeders who are sometimes relatives of the breeders (Napper & Hatchwell, 2016). Switches of individuals between flocks occur regularly, as do flock mergers, and there is no evidence that helping is a strategy to buy membership of a winter flock by birds that have failed to produce their own brood (McGowan et al., 2006; Napper & Hatchwell, 2016). Flock members forage together and gain thermoregulatory benefits at night from roosting in linear huddles (Hatchwell et al., 2009). Previous research suggests that dominance (predicted by sex and body size), rather than kinship, determines the position of individuals in linear huddles and thus the thermoregulatory benefits of roosting (McGowan et al., 2006; Napper et al., 2013). Napper et al. (2013) also showed that kinship fails to predict dominance hierarchy within flocks. Increased offspring production, and an attendant increase in kinship to the non‐breeding flock, is thus unlikely to benefit a breeder in terms of enhancing their position within a roost. There could, however, be survival benefits not yet uncovered of entering a flock with more individuals from your successful nest who are all likely to be relatives. Kinship, for example, positively affects the strength of social associations between individuals in non‐breeding flocks (Napper & Hatchwell, 2016). Reduced competition over resources, reduced predation risk or other benefits due to these stronger associations may therefore provide survival benefits to offspring and parents leading to co‐dependent survival. If producing more offspring does indeed bring survival benefits for breeders, then despite the lack of direct evidence of the presence of helpers boosting breeder survival, helpers may actually augment breeder survival indirectly through the increased production of recruits (Walters & Garcia, 2016).

### 4.4 Ageing patterns of helping behaviour

Age is a principal cause of variation in helping behaviour across cooperatively breeding species. In the majority of cooperative bird species, offspring delay dispersal to remain in their natal territory and help their parents. In these systems, helping is therefore biased towards younger age classes, while older individuals specialise on reproduction (Koenig & Dickinson, 2004). A similar bias is seen in other taxa; in stenogastrine wasps *Liostenogaster flavolineata*, for example, Field et al. (2006) showed that older individuals work less hard in an age‐based queue of inheritance. However, the reverse situation is also seen: in both apostlebirds *Struthidea cinereal* and Damaraland mole rats *Fukomys damarensis*, investment in cooperation increases, rather than decreases, with age (Woxvold et al., 2006; Zöttl et al., 2016). An individual's incentive to help will be driven by their possible returns from the help and the effects on their future fitness. As individuals age, direct fitness opportunities may be reduced due to reproductive competition with younger individuals, favouring menopause and female abstention from reproduction in species such as humans or killer whales *Orcinus orca* where intra‐group relatedness is high and indirect fitness can be gained (Cant & Johnstone, 2008; Johnstone & Cant, 2010). Alternatively, direct fitness opportunities may increase with age, for example when dominant breeders are usually older individuals. In these systems, such as meerkats *Suricata suricatta* or stenogastrine wasps (Field et al., 2006; Sharp & Clutton‐Brock, 2010), we may expect helping behaviour to decline as individuals age.

In the redirected helping system of long‐tailed tits, individuals only help if they fail in their own breeding attempts (Hatchwell, 2016). No direct benefits of helping have been detected (Meade & Hatchwell, 2010; this study), and therefore helping behaviour can be fully understood with respect to returns from indirect fitness (Hatchwell et al., 2014). We found no evidence of age effects on helping. Rather, indirect fitness accrual and the decision to help appear to be better predicted by differences in individual quality. ALR was significantly correlated with accrual of indirect fitness and a male's decision to help, supporting evidence that helping is condition dependent (McGowan et al., 2003). Although ALR was not a significant predictor for whether females will help, we found evidence that a female's decision was significantly correlated with her RE in that year. Given that all individuals in the analysis had the opportunity to help, that is a first‐order relative with a brood was available in the population, it raises the question why individuals that expended little RE would not choose to help. This may point to evidence of condition‐dependent helping in females. The females that had a larger RE and then decided to help could do so because they were of intrinsically higher quality.

Although we found no evidence here, a prospective helper's decision to help may also be influenced by the age of recipients. Where helping yields significant indirect fitness benefits to helpers, as it does in long‐tailed tits, Hamilton's (1964) rule suggests, all else being equal, that greater relatedness between actor and recipient will lead to greater returns for the actor. Potential recipients can vary in other ways, however, that may determine the benefit they will receive from being helped. Having helpers when young may aid inexperienced breeders (Magrath, 2001), while receiving help when old may alleviate greater costs of reproduction due to senescence (Hammers et al., 2019). Helpers may not, therefore, value all relatives equally if their phenotypic differences affect the benefits a helper can gain (Schulman & Rubenstein, 1983).

## 5 CONCLUSIONS ‐ INDIVIDUAL HETEROGENEITY EXPLAINS AGEING PATTERNS IN A COOPERATIVE BREEDER

Studies are accumulating that suggest sociality may play a role in explaining variation in life span across animals (Lucas & Keller, 2020). Although rare across species, the nature of the facultative redirected helping system in long‐tailed tits allowed us to separate age‐related changes in direct and indirect fitness and also facilitated a specific test for consequences of age on the decision of individuals to help. Our findings especially highlight that differences in individual quality within populations, especially in those where the risk of extrinsic mortality is extremely high, are of paramount importance when investigating ageing patterns of survival, reproduction and helping. In wild populations, the occurrence of senescence can be hard to detect (Nussey et al., 2008) and requires longitudinal datasets following many individuals. Often, senescence is revealed only when the number of sampled individuals who live substantially longer accumulates. In long‐tailed tits, these individuals seem to be of intrinsically higher quality, allowing them to escape the trade‐offs between survival and reproduction, and not fall victim to their old age.

## AUTHORS' CONTRIBUTIONS

B.J.H. managed the field study and collected data on which analyses are based over 25 years; M.R., N.J.S. and J.P.G. compiled and analysed data; M.R. wrote the manuscript along with N.J.S., B.J.H. and J.P.G. revised and edited the manuscript. All authors gave final approval for publication.

## CONFLICT OF INTEREST

The authors declare no conflict of interest.

## Supporting information


Figure S1

Data S1

## Data Availability

Data available from the Dryad Digital Repository https://doi.org/10.5061/dryad.g79cnp5rz (Roper et al., 2022).
