# Supplementary figures and images for: Individual variation explains ageing patterns in a cooperatively breeding bird, the long‐tailed tit Aegithalos caudatus
Source: J Anim Ecol. 2022 May 24;91(7):1521–34. doi: 10.1111/1365-2656.13741 (PMC9542241; doi:10.1111/1365-2656.13741)

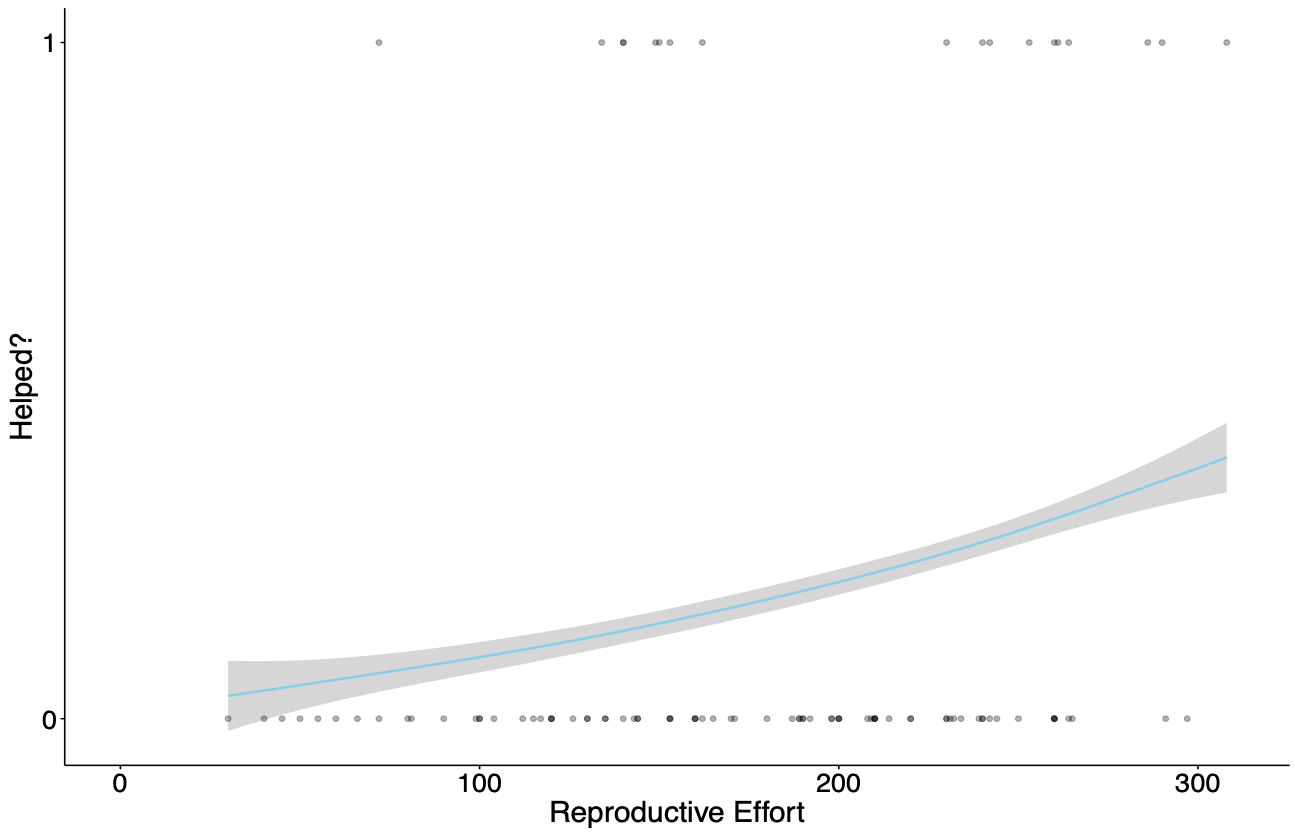

Supplement: Supplementary file 1 — Figure S1 [file JANE-91-1521-s002.png]
