# Supplementary material for: Individual variation explains ageing patterns in a cooperatively breeding bird, the long‐tailed tit Aegithalos caudatus
Source: J Anim Ecol. 2022 May 24;91(7):1521–34. doi: 10.1111/1365-2656.13741 (PMC9542241; doi:10.1111/1365-2656.13741)
Supplement: Supplementary file 2 — Data S1 [file JANE-91-1521-s001.docx]

This file contains Supporting Information for “**Individual variation explains aging patterns in a cooperatively breeding bird, the long-tailed tit (*Aegithalos caudatus*)”**

**Authors:** Mark Roper*, Nicole J. Sturrock, Ben J. Hatchwell & Jonathan P. Green

***Corresponding author**

Mark Roper

Department of Zoology, University of Oxford, 11a Mansfield Road, Oxford, OX1 3SZ, UK.

mark.roper@keble.ox.ac.uk

FILE CONTENTS

Supplementary Online Methods

Figure S1: Mean ± SE Inclusive Fitness in relation to individual age.

Figure S2: The probability a female helped in a given year as a function of their reproductive effort in that year.

Figure S3: The relationship between immigrant mass at maturity and lifetime inclusive fitness accrued

Figure S4: The effects of helpers on breeder survival as a function of brood size.

Table S2a-f: Full model outputs for the ageing patterns of breeder survival and reproduction.

Table 3: Full model outputs for the effects of helpers on breeder current and future reproduction, and survival.

References for Supplementary Online Methods

**Supplementary Online Methods**

Biometrics

Although we did not have biometric information for every year of an individual’s life, we had information on body size from at least one year for the majority of the 867 individuals for whom we had lifetime recruit production data. We tested for correlations between body mass on lifespan and lifetime inclusive fitness, accounting for sex and cohort. We performed separate models for philopatric and immigrant individuals, as body mass is measured at maturity for immigrants and the nestling stage for philopatric individuals. Since hatch date was determined through daily checks of nests, there is a 24-hour error around the estimate of nestling age. Because body mass changes rapidly during the nestling period, we chose to model body mass with tarsus length (which provides a relatively inflexible measure of age) as a covariate in order to obtain an estimate of relative body mass.

Modelling terminal age effects

As well as considering effects of age, sex and ALR on individual reproduction, there is the potential for terminal effects of age to occur, either positive (Clutton-Brock 1984) or negative (Coulson & Fairweather 2001). We were able to account for terminal investment (TI) in fledgling production and direct fitness accrual. We added a two-level class variable which categorised breeding attempts as being last (1) or not (0), and then quantified TI by comparing the difference between a birds penultimate and ultimate year of life (two data points per individual for individuals that lived for more than one year). Given that long-tailed tits attempt to breed in every year of their life, the last breeding attempt is equivalent to their final year of life. Due to this feature of long-tailed tit breeding, ALR and TI are directly correlated. This issue leads to co-linearity within models in which both variables are included, and so we analysed TI in separate models without ALR.

Extrapair paternity

We used genetic information for the social parental pair and all other ringed males present in the population to assign paternity for each nestling that was successfully genotyped between 1994 and 2019. Nestlings were excluded when we didn’t have the genotype of either the mother or social father. Paternity was checked using the likelihood-based approach implemented in CERVUS 3.0.7 (Marshall *et al.* 1998; Kalinowski *et al.* 2007), and whole broods of nestlings were excluded when paternity assignment was ambiguous for at least one nestling. By this we mean the parentage assignment was inconclusive in failing to rule out that a nestling was sired by a male other than the social male.

**Figure S1** Mean ± SE Inclusive Fitness in relation to individual age. Results are presented both for when individuals aged 4 and older are grouped as 4+ (blue), and as the raw means for age classes 4 to 7 (red). Sample sizes for each age class are displayed above each data point.

**
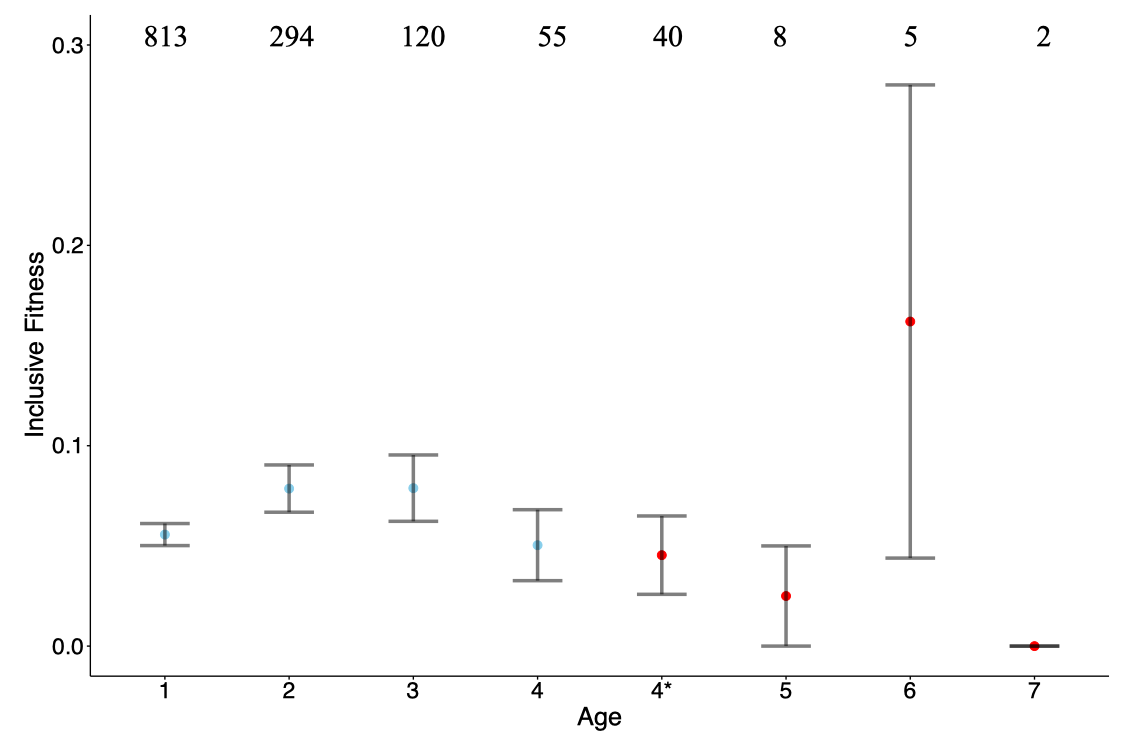
**

**Figure S2** Predicted mean ± SE probability a female helped (1) or not (0) in a breeding year when the opportunity was available, as a function of her reproductive effort in the current season.

**Figure S3** Predicted mean ± SE relationship between immigrant mass at maturity and lifetime inclusive fitness accrued. Lifetime inclusive fitness is measured as genetic offspring equivalents, stripped of the effects of helpers (See Methods for full details).


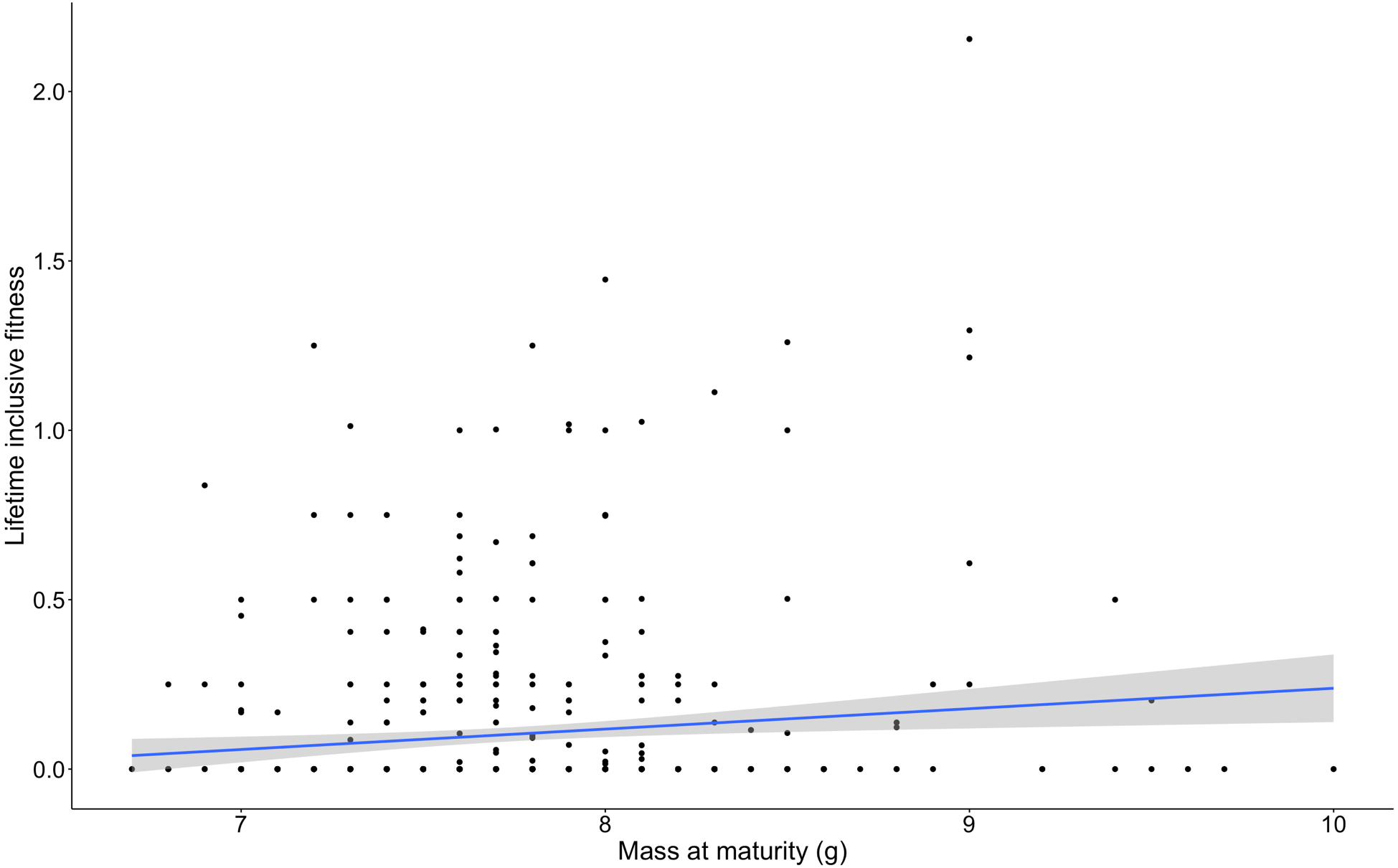


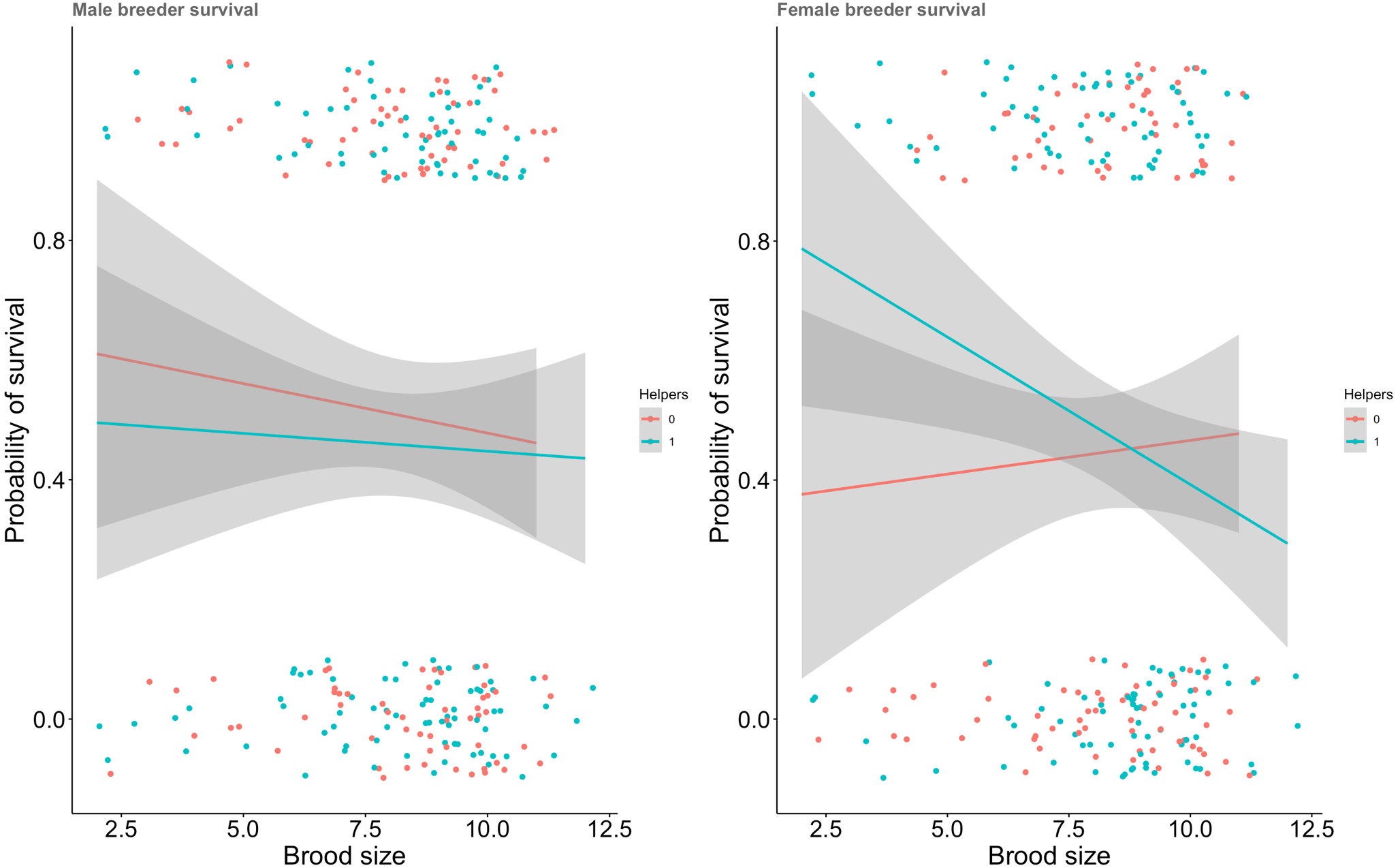
**Figure S4.** Mean ± SE predicted relationship between the presence of helpers and breeder survival as a function of brood size for male and female breeders.

**Table S2a.** **Models predicting the clutch size of a female according to its age, age at last reproduction (ALR), and whether the nest received help later in the breeding season. Random effects included were the year of study (1994-2019) and the IDs of both parents of the clutch.**

| Fixed effects | β | SE | χ^2^ | P |
| --- | --- | --- | --- | --- |
| Age | 0.04 | 0.05 | 0.76 | 0.38 |
| Age^2^ | -0.006 | 0.007 | 0.72 | 0.39 |
| ALR  Helpers | -0.004  <0.001 | 0.01  0.03 | 0.10  <0.001 | 0.75  0.99 |
|  |  |  |  |  |

Models were fitted with a poisson error structure. Significant terms are given in bold. See Methods for full details.

**Table S2b. Models predicting the number of fledglings produced by an individual according to their age, age at last reproduction (ALR), and terminal investment (TI). Random effects included were the year of study (1994-2019) and the ID of the breeder. Results are shown for all individuals assigned their true ages, and for additional analyses in which individuals aged 4 years or more are grouped as 4+ and/or individual 1978 is removed (for full details, see Methods).**

| Fixed effects | β | SE | χ^2^ | P |
| --- | --- | --- | --- | --- |
| *True age* |  |  |  |  |
| Age | 0.21 | 0.29 | 0.62 | 0.43 |
| Age^2^ | -0.06 | 0.06 | 1.49 | 0.22 |
| ALR  Sex: Male  Age:Sex | 0.15  -0.18  -0.54 | 0.07  0.13  0.50 | 4.40  2.26  1.20 | **0.04**  0.13  0.27 |
| Age^2^:Sex | 0.13 | 0.10 | 1.62 | 0.20 |
| *Grouped as 4+*  Age  Age^2^  ALR  Sex: Male  *Grouped as 4+; without 1978*  Age  Age^2^  ALR  Sex: Male  *Terminal investment*  Age  Sex: Male  TI | 0.54  -0.15  0.15  -0.18  0.70  -0.19  0.15  -0.19  -0.05  -0.20  -0.03 | 0.41  0.09  0.07  0.13  0.42  0.10  0.07  0.13  0.10  0.17  0.20 | 1.92  2.76  4.46  2.23  2.98  4.28  4.40  2.48  0.27  1.37  0.03 | 0.17  0.10  **0.03**  0.14  0.08  **0.04**  **0.04**  0.12  0.60  0.24  0.87 |
|  |  |  |  |  |

Models were fitted with a compound Poisson error structure. Significant (p < 0.05) terms are given in bold.

**Table S2c. Models predicting direct (DF) fitness accrued by an individual according to their age, age at last reproduction (ALR), and terminal investment (TI). Random effects included were the year of study (1994-2019) and the ID of the breeder. Results are shown for all individuals assigned their true ages, and for additional analyses in which individuals aged 4 years or more are grouped as 4+ and/or individual 1978 is removed (for full details, see Methods).**

| Fixed effects | β | SE | χ^2^ | P |
| --- | --- | --- | --- | --- |
| *True age* |  |  |  |  |
| Age | 0.27 | 0.32 | 0.82 | 0.36 |
| Age^2^ | -0.07 | 0.06 | 1.72 | 0.19 |
| ALR | 0.22 | 0.11 | 4.36 | **0.04** |
| Sex: Male  Age:Sex | -0.12  -0.81 | 0.14  0.76 | 0.87  1.28 | 0.35  0.26 |
| Age^2^:Sex  *Grouped as 4+*  Age  Age^2^ | 0.23  0.79  -0.20 | 0.17  0.49  0.11 | 2.10  2.98  3.85 | 0.15  0.08  **0.05** |
| ALR  Sex: Male  *Grouped as 4+; without 1978*  Age  Age^2^  ALR  Sex: Male  *Terminal investment*  Age  Sex  TI | 0.21  -0.13  0.79  -0.21  0.21  -0.15  0.09  -0.04  -0.46 | 0.11  0.14  0.52  0.12  0.12  0.14  0.16  0.23  0.30 | 4.30  1.07  2.66  3.46  3.90  1.23  0.43  0.04  3.00 | **0.04**  0.30  0.10  **0.05**  **0.04**  0.27  0.50  0.84  0.08 |
|  |  |  |  |  |

Models were fitted with a compound Poisson error structure. Significant (p < 0.05) terms are given in bold..

**Table S2d. Models predicting Inclusive fitness (IF) accrued by an individual according to their age, age at last reproduction (ALR), and terminal investment (TI). Random effects included were the year of study (1994-2019) and the ID of the breeder. Results are shown for all individuals assigned their true ages, and for additional analyses in which individuals aged 4 years or more are grouped as 4+ and/or individual 1978 is removed (for full details, see Methods).**

| Fixed effects | β | SE | χ^2^ | P |
| --- | --- | --- | --- | --- |
| *True age* |  |  |  |  |
| Age | 0.25 | 0.31 | 0.75 | 0.39 |
| Age^2^ | -0.08 | 0.06 | 2.02 | 0.16 |
| ALR | 0.30 | 0.10 | 10.93 | **<0.001** |
| Sex: Male  Age:Sex  Age^2^:Sex | 0.10  -0.06  0.04 | 0.14  0.66  0.14 | 0.54  0.01  0.12 | 0.46  0.92  0.73 |
| *Grouped as 4+*  Age  Age^2^ | 0.70  -0.19 | 0.47  0.11 | 2.45  3.56 | 0.12  0.06 |
| ALR  Sex: Male  *Grouped as 4+; without 1978*  Age  Age^2^  ALR  Sex: Male  *Terminal investment*  Age  Sex  TI | 0.29  0.09  0.79  -0.22  0.29  0.08  0.11  0.04  -0.55 | 0.09  0.14  0.49  0.11  0.10  0.14  0.14  0.23  0.27 | 10.54  0.44  2.91  4.28  10.05  0.39  0.75  0.04  4.59 | **0.001**  0.51  0.09  **0.04**  **0.002**  0.53  0.39  0.84  **0.03** |
|  |  |  |  |  |

Models were fitted with a compound Poisson error structure. Significant (p < 0.05) terms are given in bold.

**Table S2e. Models investigating the effect of age on extrapair paternity (EPP) in females and males. For females, we modelled the probability that a female sired at least one nestling that was not the genetic offspring of the social father. For males, we modelled the probability that the social father was cuckolded. Age at last reproduction (ALR) and brood size (N) were also included as fixed effects. Year and bird IDs were included as random effects.**

| Fixed effects | β | SE | χ^2^ | P |
| --- | --- | --- | --- | --- |
| *Females* |  |  |  |  |
| Age | 0.65 | 0.55 | 1.47 | 0.22 |
| Age^2^ | -0.13 | 0.10 | 1.82 | 0.18 |
| ALR | 0.004 | 0.14 | 0.001 | 0.97 |
| N | 0.002 | 0.06 | 0.001 | 0.97 |
|  |  |  |  |  |
| *Males: probability of losing EPP* |  |  |  |  |
| Age | -0.43 | 0.45 | 1.02 | 0.31 |
| Age^2^  ALR  N  *Males: probability of gaining EPP*  Age  Age^2^  ALR | 0.10  -0.07  0.01  -0.90  0.24  -0.09 | 0.07  0.14  0.07  1.03  0.20  0.52 | 2.26  0.24  0.03  0.85  1.90  0.03 | 0.13  0.63  0.86  0.36  0.17  0.86 |
|  |  |  |  |  |
|  |  |  |  |  |
|  |  |  |  |  |

Models were fitted with a binomial error structure. Significant (p < 0.05) terms are given in bold.

**Table S2f. Models predicting the probability of survival of adult long tailed tits according to the individual’s age, sex, direct fitness and reproductive effort (RE) in a given year. Mean daily rainfall (mm) over winter was also included as a fixed effect. Random effects were the year of study (1995-2018) and the ID of the individual. Results are presented for the whole population (without RE as a covariate) and separately for females and males for whom RE measures were available (for full details, see Methods).**

| Model and fixed effects | β | SE | χ^2^ | P |
| --- | --- | --- | --- | --- |
| *Whole population* |  |  |  |  |
| Age | 0.05 | 0.22 | 0.05 | 0.81 |
| Age^2^ | -0.02 | 0.04 | 0.29 | 0.59 |
| Sex | 0.20 | 0.12 | 2.99 | 0.08 |
| Direct fitness | 0.78 | 0.34 | 5.51 | **0.02** |
| Winter rainfall | 0.23 | 0.41 | 0.34 | 0.56 |
| Age:Sex  Age^2^:Sex  *Grouped as 4+*  Age  Age^2^  Sex  Direct fitness  Winter rainfall | -0.27  0.02  0.36  -0.11  0.20  0.91  -0.22 | 0.44  0.09  0.36  0.08  0.12  0.32  0.14 | 0.36  0.07  0.99  1.71  2.85  8.12  2.45 | 0.55  0.79  0.32  0.19  0.09  0.004  0.12 |
|  |  |  |  |  |
| *Females*  Age | 0.69 | 0.49 | 2.35 | 0.13 |
| Age^2^ | -0.15 | 0.11 | 2.58 | 0.11 |
| RE | 0.09 | 0.11 | 0.60 | 0.44 |
| Direct fitness | 1.45 | 0.51 | 8.56 | **0.003** |
| Winter rainfall | -0.73 | 0.28 | 5.54 | **0.02** |
|  |  |  |  |  |
| *Males*  Age | -0.01 | 0.47 | 0.001 | 0.98 |
| Age^2^ | -0.006 | 0.10 | 0.003 | 0.96 |
| RE | 0.06 | 0.12 | 0.23 | 0.63 |
| Direct fitness  Winter rainfall | -0.10  -0.23 | 0.58  0.33 | 0.027  0.48 | 0.87  0.49 |

Models were fitted with a binomial error structure. Significant terms are given in bold.

**Table S3. Models investigating the effect of helpers on breeder age-trajectories of future reproduction in year n+1. We included fixed effects of age (linear and quadratic), brood size, relative fledge date (to 1^st^ May), winter rainfall and spring temperature, whether the individual produced recruits or not and the presence of helpers. Random effects included in all models were individual ID and Year.**

| Fixed effects | β | SE | χ^2^ | P |
| --- | --- | --- | --- | --- |
| *Clutch Size*  Age in year n+1  Age^2^ in year n+1  Helpers in year n  ALR  Brood size year n  Recruits in year n  Relative fledge date year n  Spring temperature  Winter rainfall  *Lay Date*  Age in year n+1  Age^2^ in year n+1  Helpers in year n  ALR  Brood size year n  Recruits in year n  Relative fledge date year n  Spring temperature  Winter rainfall  *Fledglings* | 0.03  -0.005  -0.01  -0.003  0.001  0.06  0.001  -0.001  0.01  3.70  -0.55  -0.56  -0.13  -0.51  -0.11  0.15  -2.27  3.19 | 0.28  0.04  0.09  0.04  0.02  0.10  0.005  0.03  0.09  2.54  0.37  1.00  0.50  0.23  1.13  0.06  0.63  2.26 | 0.02  0.02  0.02  0.07  <0.001  0.34  0.05  0.021  0.02  2.13  2.33  0.31  0.07  4.83  0.002  6.14  11.98  2.22 | 0.93  0.90  0.89  0.93  0.99  0.56  0.83  0.96  0.89  0.14  0.13  0.58  0.79  **0.03**  0.96  **0.01**  **0.001**  0.14 |
| Age in year n+1  Age^2^ in year n+1  Helpers in year n  ALR  Brood size year n  Recruits in year n  Relative fledge date year n  Spring temperature  Winter rainfall  *Direct Fitness*  Age in year n+1  Age^2^ in year n+1  Helpers in year n  ALR  Brood size year n  Recruits in year n  Relative fledge date year n  Spring temperature  Winter rainfall | -0.93  0.17  -0.05  -0.03  0.01  0.36  -0.01  0.08  0.13  -1.76  0.27  0.18  0.08  0.02  -0.28  0.001  0.29  0.59 | 0.92  0.13  0.31  0.15  0.08  0.33  0.02  0.10  0.37  1.55  0.22  0.47  0.24  0.11  0.54  0.03  0.19  0.56 | 1.06  1.70  0.03  0.05  0.03  1.21  0.33  0.61  0.12  1.81  2.29  0.14  0.10  0.02  0.31  0.08  3.54  1.26 | 0.30  0.19  0.87  0.82  0.87  0.27  0.56  0.44  0.72  0.18  0.13  0.71  0.75  0.88  0.58  0.78  0.06  0.26 |

Clutch size and relative lay date were modelled with a gaussian error structure, while survival, fledgling production and direct fitness were modelled with binomial error structure. Significant (p < 0.05) terms are given in bold.

**References**

1. Tarwater, C.E. & Arcese, P. (2017). Age and years to death disparately influence reproductive allocation in a short-lived bird. *Ecology, 98,* 2248-2254.
2. Hamilton, W.D. The genetical evolution of social behaviour. *J. Theor. Biol, 7,* 1-16 (1964).
3. Green, J.P. & Hatchwell, B.J. (2018). Inclusive fitness consequences of dispersal decisions in a cooperatively breeding bird, the long-tailed tit (*Aegithalos caudatus*). *Proceeding of the National Academy of Sciences. USA, 115,* 12011-12016.
4. Marshall TC, Slate J, Kruuk LEB, Pemberton JM (1998) Statistical confidence for likelihood-based paternity inference in natural populations. *Mol Ecol, 7,* 639–655.
5. Kalinowski ST, Taper ML, Marshall TC (2007) Revising how the computer program CERVUS accommodates genotyping error increases success in paternity assignment. *Mol Ecol,* *16,* 1099–1106.
6. Clutton-Brock, T. (1984). Reproductive effort and terminal investment in iteroparous animals. *Am. Nat*, *123,* 212–229.
7. Coulson J.C.& Fairweather J.A. (2001). Reduced reproductive performance prior to death in the black-legged kittiwake: senescence or terminal illness?. *J. Avian Biol,  32,* 146–152.
